# Perioperative Renal Ultrasonography of Arterio-to-Venous Coupling Predicts Postoperative Complications after Major Laparoscopic Urologic Surgery

**DOI:** 10.3390/jcm12155013

**Published:** 2023-07-30

**Authors:** Claudia Brusasco, Guido Tavazzi, Giada Cucciolini, Pierpaolo Di Nicolò, Adrian Wong, Antonia Di Domenico, Federico Germinale, Federico Dotta, Marco Micali, Federico Coccolini, Gregorio Santori, Federico Dazzi, Carlo Introini, Francesco Corradi

**Affiliations:** 1 Anaesthesia and Intensive Care Unit, E.O. Ospedali Galliera, Mura della Cappuccine 14, 16128 Genoa, Italy; marco.micali@galliera.it; 2 Unit of Anesthesia and Intensive Care, Department of Clinical-Surgical, Diagnostic and Pediatric Sciences, University of Pavia, 27100 Pavia, Italy; guido.tavazzi@unipv.it; 3 Department of Surgical, Medical, Molecular Pathology and Critical Care Medicine, University of Pisa, 56126 Pisa, Italy; giada.cucciolini@phd.unipi.it (G.C.); fededazzi92@gmail.com (F.D.); 4 Nephrology and Dialysis Unit, S. Maria della Scaletta Hospital, 40026 Imola, Italy; p.dinicolo@hotmail.it; 5 Department of Critical Care, King’s College Hospital, London SE5 9RS, UK; avkwong@mac.com; 6 Urology Unit, E.O. Ospedali Galliera, 16128 Genoa, Italy; antonia.didomenico@galliera.it (A.D.D.); federico.germinale@galliera.it (F.G.); federico.dotta@galliera.it (F.D.); carlo.introini@galliera.it (C.I.); 7 General, Emergency and Trauma Surgery, Pisa University Hospital, 56100 Pisa, Italy; federico.coccolini@unipi.it; 8 Department of Surgical Sciences and Integrated Diagnostics (DISC), University of Genoa, 16132 Genoa, Italy; gregorio.santori@unige.it

**Keywords:** arterio-venous coupling, Doppler, perfusion, intra-abdominal pressure, pneumoperitoneum, neuromuscular blockade, acute kidney injury, enhanced recovery after surgery, urologic surgery, POCUS

## Abstract

Point-of-care ultrasonography (POCUS) with concomitant venous and arterial Doppler assessment enables clinicians to assess organ-specific blood supply. To date, no studies have investigated the usefulness of including a comprehensive perioperative POCUS assessment of patients undergoing major laparoscopic surgery. The primary aim of the present study was to evaluate whether the combined venous and arterial renal flow evaluation, measured at different time points of perioperative period, may represent a clinically useful non-invasive method to predict postoperative acute kidney injury (AKI) after major laparoscopic urologic surgery. The secondary outcome was represented by the development of any postoperative complication at day 7. We included 173 patients, subsequently divided for analysis depending on whether they did (n = 55) or did not (n = 118) develop postoperative AKI or any complications within the first 7 days. The main results of the present study were that: (1) the combination of arterial hypoperfusion and moderate-to-severe venous congestion inferred by POCUS were associated with worst outcomes (respectively, HR: 2.993, 95% CI: 1.522–5.884 and HR: 8.124, 95% CI: 3.542–18, *p* < 0.001); (2) high intra-operative abdominal pressure represents the only independent determinant of postoperative severe venous congestion (OR: 1.354, 95% CI: 1.017–1.804, *p* = 0.038); (3) the overall number of complications relies on the balance between arterial inflow and venous outflow in order to ensure the adequacy of peripheral perfusion; and (4) the overall reliability of splanchnic perfusion assessment by Doppler is high with a strong inter-rater reliability (ICC: 0.844, 95% CI: 0.792–0.844). The concomitant assessment of arterial and venous Doppler patterns predicts postoperative complications after major laparoscopic urologic surgery and may be considered a useful ultrasonographic biomarker to stratify vulnerable patients at risk for development of postoperative complications.

## 1. Introduction

Perioperative AKI is a serious complication associated with high morbidity and mortality [1], occurring in 30–40% of all major surgical interventions [2,3]. Even after complete recovery from AKI, patients may develop chronic or end-stage renal disease [4], with an up to 12.6-fold relative risk of death [5]. Moreover, not only is mortality higher, but also non-renal post-surgical complications are more frequent in these patients [6,7].

While close attention is generally paid to pre- and postoperative management, the role of surgical techniques and perioperative management in the development of AKI and other complications remains underappreciated.

Postoperative AKI may be due to either sudden arterial hypoperfusion causing acute tubular necrosis, or renal venous congestion determined by intraluminal hypertension. These two mechanisms can lead to a dramatic fall in glomerular filtration rate; in fact, the net ultrafiltration pressure is derived from the opposing forces of the blood hydrostatic pressure in the glomerulus versus the combination of capsular hydrostatic pressure and blood colloid osmotic pressure [8].

Point-of-care ultrasonography (POCUS) with arterial Doppler assessment enables clinicians to assess organ-specific blood supply in both arteries and veins [9,10]. Recently, Doppler patterns of intrarenal venous flow (IRVF) have been described to predict acute kidney injury (AKI) and post-surgical complications after open heart surgery [11]. However, to date, no studies have investigated the usefulness of including IRVF in a perioperative POCUS assessment of patients undergoing major laparoscopic surgery.

Our hypothesis is that not only arterial perfusion but even venous drainage may influence organ perfusion, determining AKI and other postoperative complications. The primary aim of the present study is to evaluate whether, in patients undergoing major laparoscopic urologic surgery, the combination of IRVF and renal Doppler resistive index (RDRI) measured at different time points of perioperative period represents a clinically useful non-invasive method to predict postoperative AKI (within the first seven days) [12]. The secondary aim is to evaluate if the IRVF and RDRI can predict the occurrence of any other postoperative complication at day seven.

## 2. Methods

This is an observational, monocentric, prospective study. The study was approved by the local ethics committee (CER Liguria: 7/2019-id: 4378) and all the enrolled patients gave written informed consent to the use of their data.

### 2.1. Patients

Between December 2019 and April 2022, all the adult patients admitted for major laparoscopic surgery to the urology section of the Galliera Hospital, Genova, Italy, were evaluated for enrollment.

Inclusion criteria were: age > 18 aa, the ability to provide informed consent, and availability of the operators to perform a POCUS assessment before surgery.

Patients were excluded if they had one or more of the following: inability to sample Doppler indices, severe chronic kidney disease (estimated glomerular filtration rate < 15 mL/min per 1.73 m^2^ or dialysis), renal transplantation (planned or received), critical preoperative state (defined as preoperative mechanical ventilation, vasopressor or inotropic support), emergency surgery, obstructive uropathy, urinary infection, or sepsis (Supplemental Figure S1).

### 2.2. Data Collection

Demographic and baseline clinical data were collected including the Charlson comorbidity index (CCI), the Karnofsky scale, and the preoperative American Society of Anesthesiologists Classification (ASA score). Creatinine measurements were obtained before surgery and daily after surgery until discharge. The estimated glomerular filtration rate was calculated using the Modified Diet in Renal Disease formula [13]. AKI was defined accordingly to the Kidney Disease Improving Global Outcomes criteria, based on creatinine values and urinary output variations [14]. Intraoperative, clinical, and laboratory data were monitored until hospital discharge.

### 2.3. Renal Ultrasound Assessment

All ultrasonographic examinations were performed by two expert operators using a Mindray TE7 ultrasound device (Shenzhen Mindray Bio-Medical Electronics Co., Shenzhen, China) with a 2.5–5.0 MHz convex array transducer for kidney’s examination with the patient in a supine position [15,16]. After a general preliminary examination of the abdominal cavity and organs, Doppler ultrasound measurements were obtained along the interlobar vessels of the renal cortex.

The ultrasound examination was considered technically acceptable if the following criteria were met: (a) the possibility to obtain a clear two-dimensional longitudinal scan with definition of renal parenchyma, (b) a good color Doppler image with visualization of IRVF was feasible, and (c) at least three consecutive Doppler time-velocity spectra were obtained. Pulsed-wave Doppler spectrum was increased by using the lowest frequency-shift range that did not cause aliasing, and the wall filter was set at a low frequency (100 MHz). RDRI was calculated according to Planiol and Pourcelot’s protocol as (VMax–VMin)/VMax [17]. The value of Doppler measurements for IRVF was calculated and averaged to minimize sampling error. The IRVF patterns were classified as continuous, biphasic, or monophasic, depending on their presence during the whole heart cycle, intermittency with a systolic and a diastolic peak, or the only presence of a diastolic peak [18].

All Doppler examinations were recorded on the day before surgery, at postoperative day 1, and at hospital discharge. The attending physician was unaware of the results of the ultrasound examinations. The investigators were blinded to the clinical parameters while performing the ultrasound assessments.

### 2.4. Statistical Analysis

All the variables are expressed as median and interquartile range (IQR) for continuous variables, or as percentage (%) for categorical variables. The Shapiro–Wilk test was used to evaluate the normal distribution of continuous variables. The Mann–Whitney U-test or Fisher’s exact test were used to evaluate differences between groups for continuous or categorical variables, respectively. Cumulative probability for lack of postoperative AKI or any complications in patients with different Doppler profiles were calculated with the Kaplan–Meier product-limit estimator. The censored/uncensored patients corresponded to hospital discharge/occurrence of complications. The log-rank (Mantel–Cox) test was applied to evaluate the difference between Kaplan–Meier curves in Doppler profiles [19].

The main Doppler and clinical variables were entered into a Cox proportional hazard analysis. The variables associated in univariate models with occurrence of postoperative AKI or any complications with a *p* < 0.10 without violating the assumption of proportional risk were entered into a multivariate Cox proportional hazard model. In each Cox model both the regression coefficient and hazard ratio (HR) with 95% confidence interval (CI) were calculated. Logistic regression was used to identify independent variables potentially associated with IRVF patterns identified as predictors of complications, by providing odds ratio (OR) with the corresponding 95% CI for each model. The receiver operating characteristic (ROC) curves were used to determine the best cut-off values. The area under the ROC curve (AUC) and the related 95% CI were calculated. Linear forward regression models with the Bayesian information criterion were also performed to evaluate the relationship between independent variables and monophasic IRVF assumed as the dependent variable. Inter-observer variability was tested by intraclass correlation coefficient (ICC) in two-way models for agreement (strength of absolute agreement between raters was considered poor, fair, moderate, strong, or almost perfect according to ICC values < 0.30, 0.3–0.49, 0.50–0.69, 0.70–0.89, and ≥0.90, respectively).

Statistical significance was assumed at *p* < 0.05 with a two-tailed null hypothesis. The statistical analyses were carried out by using SPSS (version 27.0; SPSS Inc., Chicago, IL, USA) and R environment (version 4.0.3; R Foundation for Statistical Computing, Vienna, Austria).

## 3. Results

A total of 200 patients met the inclusion criteria. After exclusion criteria, 173 patients were included in the analysis (Table 1) and divided into two groups, depending on whether they did (n = 55) or did not (n = 118) develop postoperative complications within the first 7 days. The demographics and clinical characteristics of the patients are illustrated in Table 1.

### 3.1. Renal Doppler Assessment

**Figure 1:**
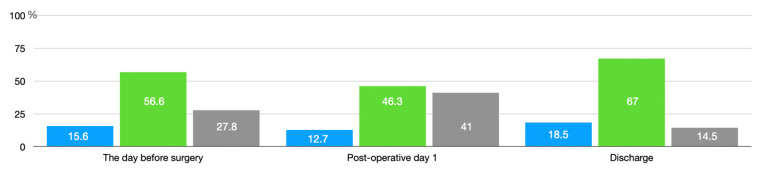
Intrarenal venous flow pattern distribution during hospitalization. In blue: continuous venous flow pattern; in green: biphasic venous flow pattern; in gray: monophasic venous flow pattern.

The distribution of the morphology of the IRVF pattern is illustrated in Figure 1. Before surgery, the IRVF pattern (Figure 1) was continuous in 27 patients (15.6%), biphasic in 98 (56.6%), and monophasic in 48 (27.7%). The median RDRI was 0.65 (IQR 0.58–0.70).

On postoperative day 1, the IRVF pattern was continuous in 22 patients (12.7%), biphasic in 80 (46.2), and monophasic in 71 (41%). The median RDRI was 0.69 (IQR 0.63–0.74). As compared with preoperative measurements, the IRVF pattern worsened in 54 patients (31%), changing from continuous to biphasic in 10, from continuous to monophasic in 8, and from biphasic to monophasic in 36, suggestive of a trend toward stages of more severe congestion.

At hospital discharge, the IRVF pattern was continuous in 32 patients (18.5%), biphasic in 116 (67.1%), and monophasic in 25 (14.5%). The median RDRI was 0.66 (IQR 0.59–0.70). Overall, the IRVF pattern improved from the pre- to postoperative period in 29 patients and remained unchanged in 90, while RDRI significantly increased (0.65 (IQR 0.58–0.70) vs. 0.69 (0.63–0.74) *p* < 0.001).

### 3.2. Postoperative AKI

Patients who developed postoperative AKI had more cardiovascular comorbidities treated by oral β-blockers with slightly higher baseline RDRI, higher intra-abdominal pressure due to surgical pneumoperitoneum, lower renal filtration gradient, and lower hemoglobin levels in the postoperative period (Table 2).

**Figure 2:**
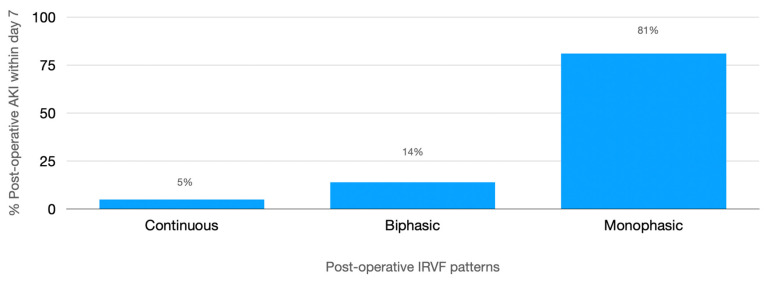
Prevalence of different postoperative intrarenal venous flow patterns and incidence of postoperative AKI.

In addition, they had more frequent IRVF worsening with a higher incidence of postoperative monophasic pattern (40% vs. 23% *p* < 0.001) (Figure 2) and higher postoperative RDRI (0.73 (0.67–0.75) vs. 0.67 (0.62–0.73) *p* < 0.001) with the best cut-off of 0.72 (AUROC 0.67, CI: 0.58–0.77, *p* < 0.001). Inferior vena cava maximum diameter and collapsibility index were not significantly different between the two groups (Table 2).

**Figure 3:**
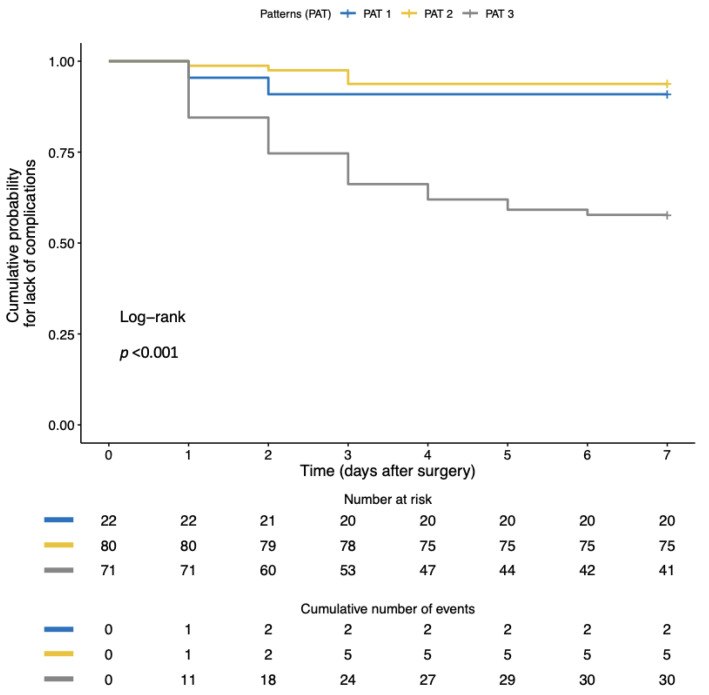
Comparison of the Kaplan–Meier curves for lack of adverse outcomes (postoperative AKI) in patients with continuous (yellow line), biphasic (blue line) or monophasic (gray line) intra-renal venous flow pattern (IRVF). Patients were censored at postoperative day 7. Kaplan–Meier analysis showed a significantly shorter time to first complication with monophasic than non-monophasic IRVF patterns.

In multivariate analysis, IRVF pattern and RDRI were the only factors independently associated with postoperative AKI (Table 3). Kaplan–Meier analysis showed a significantly shorter time to the first complication with monophasic than non-monophasic IRVF patterns (Figure 3).

### 3.3. Postoperative Complications

Of the 55 patients with various types of complications (Table 4), no one died or required intensive care unit admittance.

Patients who developed any complications within day 7, compared with patients who did not (Table 5), had a higher intra-abdominal pressure due to surgical pneumoperitoneum, lower abdominal perfusion pressure, lower renal filtration gradient, and a tendency for greater blood loss requiring more fluid administration in the intraoperative period with lower hemoglobin levels in the postoperative period.

**Figure 4:**
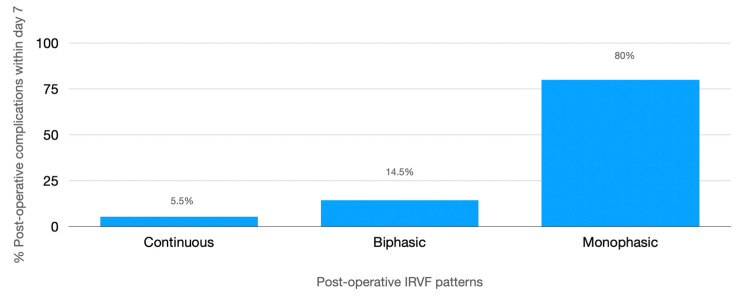
Prevalence of different postoperative intrarenal venous flow patterns and incidence of any complications.

**Figure 5:**
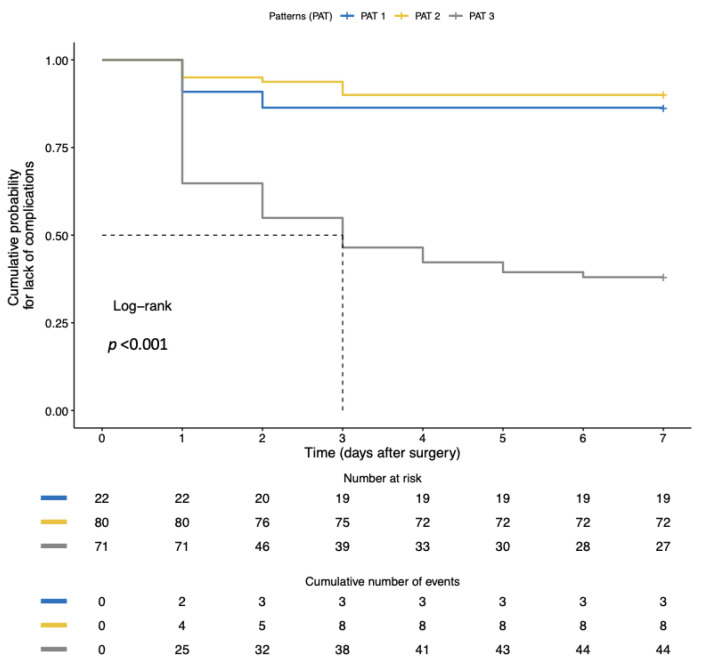
Comparison of the Kaplan–Meier curves for lack of adverse outcomes (any postoperative complications) in patients with continuous (yellow line), biphasic (blue line) or monophasic (gray line) intra-renal venous flow pattern. Patients were censored at postoperative day 7. Kaplan–Meier analysis showed a significantly shorter time to first complication with monophasic than non-monophasic IRVF patterns.

In addition, they had more frequent IRVF worsening (58% vs. 19% *p* < 0.001) (Table 5) with a higher incidence of postoperative monophasic pattern (Figure 4) and higher postoperative RDRI (0.74 (0.73–0.77) vs. 0.67 (0.61–0.71) *p* < 0.001) with the best cut-off of 0.72 (AUROC 0.73, CI: 0.65–0.81, *p* < 0.001). Inferior vena cava maximum diameter and collapsibility index were not significantly different between the two groups (Table 5). In multivariate analysis, the IRVF pattern and RDRI were the only factors independently associated with postoperative complications (Table 6). Kaplan–Meier analysis showed a significantly shorter time to the first complication with monophasic than non-monophasic IRVF patterns (Figure 5).

### 3.4. Postoperative Monophasic IRVF

At univariate analysis, the development of monophasic IRVF was associated with high pneumoperitoneum pressure and a large amount of fluid given during the intraoperative period (Table 7), but not mean arterial pressure. In the multivariate analysis, the intra-abdominal pressure remained the only factor independently associated with the development of monophasic IRVF (Table 8).

### 3.5. Arterial-to-Venous Coupling and Development of Complications

**Figure 6:**
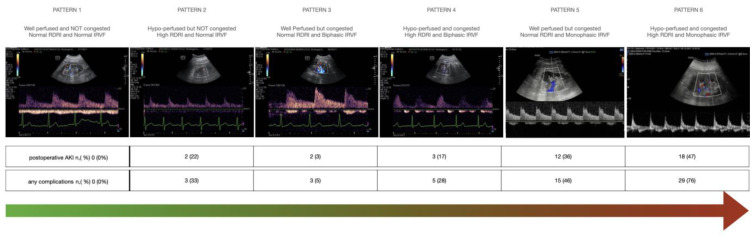
The six different combinations of arterio-to-venous coupling with the corresponding percentage of postoperative AKI or any complications at day 7.

From the present cohort, combining arterial and venous Doppler spectra, we can identify six different perfusion profiles: (1) “normal”, with preserved arterial perfusion without congestion characterized by an RDRI < 0.72 and continuous IRVF; (2) ”pure hypo-perfused” with RDRI > 0.72 and continuous IRVF; (3) “compensated congestive profile” with biphasic IRVF but RDRI < 0.72; (4) “mixed mild hypo-perfused and decompensated congestive profile” with biphasic IRVF and RDRI > 0.72; (5) “pure severe congestive profile” with monophasic IRVF but normal RDRI < 0.72; and (6) “mixed severe hypo-perfused-congestive profile” with monophasic IRVF and increased RDRI > 0.72. (Figure 6).

The frequency distribution of these profiles was associated with the occurrence of postoperative AKI or any complications (Table 9).

**Figure 7:**
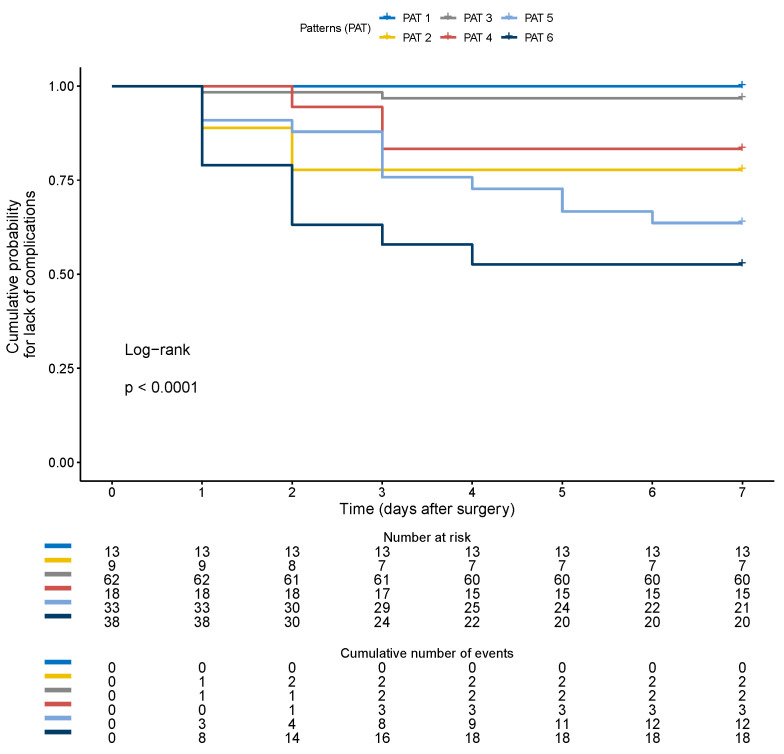
Kaplan–Meier estimates of the time to first postoperative AKI for the six flow profiles. PAT (1) “normal”, with preserved arterial perfusion without congestion characterized by RDRI < 0.72 and continuous IRVF; PAT (2) ”pure hypo-perfused” with RDRI > 0.72 and continuous IRVF; PAT (3) “compensated congestive profile” with biphasic IRVF but RDRI < 0.72; PAT (4) “mixed mild hypo-perfused and decompensated congestive profile” with biphasic IRVF and RDRI > 0.72; PAT (5) “pure severe congestive profile” with monophasic IRVF but normal RDRI < 0.72; PAT (6) “mixed severe hypo-perfused-congestive profile” with monophasic IRVF and increased RDRI > 0.72.

**Figure 8:**
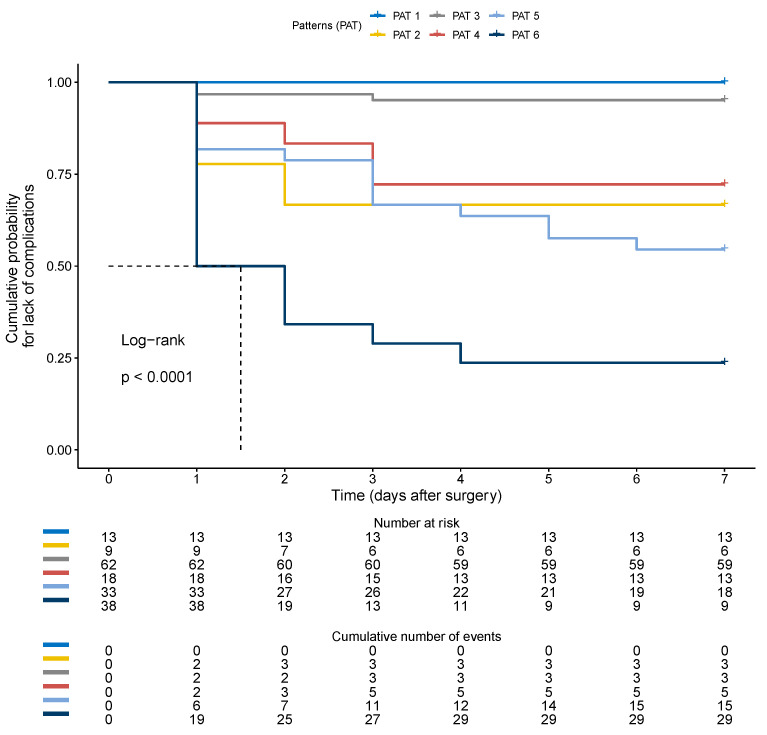
Kaplan–Meier estimates of the time to first complication for the six flow profiles. PAT (1) “normal”, with preserved arterial perfusion without congestion characterized by RDRI < 0.72 and continuous IRVF; PAT (2) ”pure hypo-perfused” with RDRI > 0.72 and continuous IRVF; PAT (3) “compensated congestive profile” with biphasic IRVF but RDRI < 0.72; PAT (4) “mixed mild hypo-perfused and decompensated congestive profile” with biphasic IRVF and RDRI > 0.72; PAT (5) “pure severe congestive profile” with monophasic IRVF but normal RDRI < 0.72; PAT (6) “mixed severe hypo-perfused-congestive profile” with monophasic IRVF and increased RDRI > 0.72.

The most frequent profile (number 3) was present in 36% of patients and associated with a low rate of complications (5%). Profiles with arterial hypoperfusion but no congestion (numbers 2 and 4) were associated with a 28–33% complication rate. Profiles with severe venous congestion were associated with complication rates from 46% in the case of isolated venous congestion to 76% in the case of concomitant arterial hypoperfusion. Kaplan–Meier estimates of the time to first postoperative AKI or complication for the six flow profiles are represented, respectively, in Figure 7 and Figure 8.

### 3.6. Reliability

The IRVF patterns were identifiable in all cases with complete inter-observer agreement.

RDRI could not be determined in one or in both kidneys of 10 subjects (5%) due to poor acoustic windows caused by intestinal gas, presence of surgical drainage or obesity. The inter-rater agreement for RDRI was strong (ICC 0.844 (95% CI from 0.792 to 0.844)).

## 4. Discussion

In this study, the prediction of AKI and other postoperative complications after major urologic laparoscopic surgery was researched using a renal point-of-care ultrasound. The main findings were that (1) a postoperative monophasic IRVF pattern predicts AKI or any complications by day 7, (2) high intraoperative abdominal pressure represents the only independent determinant of postoperative monophasic IRVF pattern, and (3) the overall number of postoperative AKI or any complication relies on the balance between arterial inflow and venous outflow in order to ensure the adequacy of peripheral perfusion.

Postoperative IRVF patterns have not been extensively studied in the perioperative period of laparoscopic surgery. This study shows that IRVF assessment is feasible with a widely available conventional ultrasonographic system and can provide information on the risk of postoperative AKI as well as any complications within the postoperative day 7. Patients who developed postoperative monophasic IRVF patterns were those who received larger amounts of intraoperative fluids and had higher intra-abdominal pressure, independently of mean arterial pressure. Although a low mean arterial pressure may represent a condition affecting intrarenal circulation, the lack of significant association with changes in IRVF patterns in the present study suggests that mechanisms other than the sole blood supply could be involved in the development of renal dysfunction. Some of these might be the increase in venous resistance to flow induced by high intra-abdominal pressure and reduction of parenchymal compliance due to increased renal interstitial pressure caused by fluid overload; both mechanisms ultimately lead to a “splanchnic tamponade” [20].

This is an important issue to be considered because introduces the concept that splanchnic tamponade due to venous congestion is not necessarily caused by fluid overload but may be due to other conditions affecting vascular compliance such as abdominal hypertension or abdominal compartment syndrome, leading to the development of the new concept of “dry” splanchnic tamponade.

Most of the ultrasonographic studies on congestion came from the application of VEXUS protocols in cardiologic patients with varying degrees of right and/or left cardiac dysfunction and concomitant fluid overload [9]. However, our results, collected in a population of elective surgical patients submitted to a strict ERAS protocol without known cardiac pathologies or signs of heart decompensation, showed postoperative Doppler signs of splanchnic congestion even in the absence of dilatation of the inferior vena cava, thus suggesting the absence of fluid overload. These findings support the hypothesis that in our patients the main pathophysiological mechanism causing congestion may be due to a “restrictive venous compliance” rather than right heart dysfunction or overt fluid overload. If our findings would be confirmed in further studies, the VEXUS ultrasound approach as originally described, should be rethought avoiding considering the inferior vena cava as “a gate” in order to decide whether or not to proceed with the assessment of congestion in the splanchnic compartment. This consideration would be extremely important in the context of critically ill patients, due to the multiple pitfalls of IVC ultrasonography in this setting [21].

Furthermore, in our study, a considerable number of patients in the postoperative period presented with venous patterns suggestive of mild-to-moderate congestion, without actually developing postoperative AKI or any complications. These findings support the hypothesis that venous congestion may be effectively counterbalanced by a proper cardiac output and arterial in-flow [22].

Moreover, the monophasic IRVF pattern was associated with worst outcomes compared with the continuous or biphasic patterns; these findings are supported by the influence of splanchnic congestion on postoperative complications [23,24,25,26] even if the amount of intraoperative fluid administered and pneumoperitoneum pressures were relatively low, according to the ERAS protocols of our center [27]. Even if differences in the intrabdominal pressures during pneumoperitoneum were statistically different between patients who eventually developed postoperative AKI or any complications compared with those who didn’t, they were not predictive of AKI or any complication and we must say that the absolute value of these pressures was very close, and thus unhelpful in the clinical practice. Conversely, postoperative AKI and any complications can be predicted by IRVF patterns in the early postoperative period, potentially allowing the timely treatment of individual patients.

RDRI was previously demonstrated to be as a useful tool to assess splanchnic regional perfusion in critically ill patients [10,15,16]. The present study confirms and expands these findings, as an impaired postoperative RDRI was independently associated with a strong odds ratio with postoperative AKI and any complications.

Our study is different from all those previously published on the topic as for the first time it simultaneously evaluates arterial perfusion, venous congestion, and their mutual interrelationship. The combination of overt arterial hypoperfusion (RDRI > 0.72) and moderate-to-severe venous congestion (biphasic or monophasic venous patterns) was associated with the worst outcomes, suggesting that only the concomitant assessment of the arterial and venous side of the circulation may provide the entire picture of the adequacy of end-organ perfusion requirements.

Hopefully, this concept will pave the way for designing a large randomized clinical trial to investigate if and how addressing the adequacy of splanchnic perfusion more precisely by US can improve the postoperative outcomes of these patients.

We acknowledge some study limitations. This is a single-center study from a referral center. It has inherent flaws related to selection and referral bias. Our work was not aimed to evaluate the different effects on splanchnic circulation by different types of urological surgery that may be important factors affecting perfusion. Cardiac output, the major determinant of splanchnic perfusion, was not measured because it does not comply with our ERAS protocol in this type of surgery. The results of the present study refer to elective laparoscopic urological surgery and therefore cannot be transposed to other types of surgery.

## 5. Conclusions

The concomitant assessment of the arterial (RDRI) and venous (IRVF) side of the circulation predicts postoperative complications after major laparoscopic urologic surgery and may be considered a useful ultrasonographic biomarker to stratify vulnerable patients at risk for the development of postoperative complications. Whether prediction of AKI and other complications can anticipate or ameliorate the treatment of perfusion disorders, improving outcomes requires further investigation.

## Figures and Tables

**Table 1:** Patient characteristics and data at hospital admission.

	All Patients (n = 173)
Demographics	
Age, y	68 (60–73)
Male, n (%)	157 (91)
Body mass index, kg/m^2^	25 (23–28)
Baseline clinical data	
Charlson Comorbidity Index	4 (2–5)
Karnofsky index	100 (90–100)
ECOG performance status scale	0 (0–0)
ASA classification	2 (2–2)
Baseline laboratory data	
Hemoglobin, g/dL	14 (13–15)
Sodium, mmol/L	140 (139–141)
Potassium, mmol/L	4.2 (4–4.5)
Urea, mg/dL	38 (31–44)
Serum Creatinine, mg/dL	0.9 (0.8–1.1)
eGFR mL/min/1.73 m^2^	83 (65–101)
Comorbidities, n (%)	
Hypertension	78 (49)
Diabetes mellitus	15 (9)
Active smoker	22 (14)
Chronic cardiovascular diseases	21 (13)
Chronic Respiratory diseases	13 (8)
Maintenance therapies	
ACEI, n (%)	31 (19)
ARB, n (%)	32 (20)
β-Blocker, n (%)	35 (22)
Loop diuretic, n (%)	20 (12)
Major laparoscopic surgery	
Radical nephrectomy, n (%)	27 (16)
Partial nephrectomy, n (%)	21 (12)
Radical prostatectomy, n (%)	112 (65)
Adrenalectomy, n (%)	2 (1)
Others, n (%)	11 (6)

**Table 2:** Comparison between patients eventually developing postoperative AKI versus those who did not.

	Postoperative AKI Group (n = 37)	No Postoperative AKI Group (n = 136)	*p*
Demographics			
Age, y	70 (62–74)	67 (59–73)	0.271
Male, n (%)	49 (89)	108 (92)	0.540
Body mass index, kg/m^2^	25 (23–29)	25 (23–28)	0.862
Baseline clinical data			
Charlson comorbidity index	5 (4–6)	4 (2–5)	0.003
ECOG performance status scale	0 (0–0)	0(0–0)	0.074
ASA classification	2 (2–2)	2 (2–2)	0.236
Baseline laboratory data			
Hemoglobin, g/dL	14 (13–15)	15 (13–16)	0.319
Sodium, mmol/L	141 (139–142)	140 (139–141)	0.575
Potassium, mmol/L	4.4 (4.1–5.0)	4.2 (4.0–4.5)	0.132
Urea, mg/dL	40 (32–50)	37 (30–44)	0.124
Serum creatinine, mg/dL	1.00 (0.90–1.20)	0.90 (0.80–1.00)	0.273
eGFR mL/min/1.73 m^2^	69 (63–89)	85 (67–104)	0.074
Comorbidities, n (%)			
Hypertension	25 (48)	53 (47)	0.449
Diabetes mellitus	7 (14)	8 (7)	0.094
Active smoker	3 (6)	19 (17)	0.418
Chronic cardiovascular diseases	9 (19)	12 (11)	0.026
Chronic respiratory diseases	5 (11)	8 (7)	0.493
Maintenance therapies			
ACEI/ARB, n (%)	24 (44)	39 (33)	0.478
β-Blocker	14 (28)	21 (19)	0.019
Loop diuretic, n (%)	5 (10)	15 (13)	0.771
Intraoperative period			
MAP, mm Hg	75 (80–85)	79 (74–85)	0.131
Heart rate, beats/min	59 (50–65)	62 (54–65)	0.149
Intra-abdominal pressure, mmHg	10 (10–12)	10 (8–12)	0.013
Abdominal perfusion pressure, mmHg	65 (60–73)	69 (63–77)	0.050
Renal filtration gradient, mmHg	55 (50–61)	60 (52–66)	0.020
Length of surgery, minutes	150 (89–171)	140 (109–180)	0.830
Length of pneumoperitoneum, minutes	120 (45–143)	116 (80–151)	0.353
Trendelemburg, degrees (°)	19 (15–24)	20 (15–24)	0.216
Blood loss, mL	200 (50–400)	200 (50–375)	0.585
Intraoperative fluids, mL	1300 (975–1700)	1200 (900–1600)	0.478
Postoperative period			
Hemoglobin, g/dL	11 (10–12)	12 (11–13)	0.009
Sodium, mmol/L	139 (137–140)	139 (137–141)	0.376
Potassium, mmol/L	4.1 (3.9–4.3)	4.1 (3.8–4.3)	0.998
Urea, mg/dL	39 (30–49)	31 (25–39)	<0.001
Postoperative creatinine, mg/dL	1.5 (1.3–2.0)	1.0 (0.8–1.1)	<0.001
eGFR mL/min/1.73 m^2^	47 (30–65)	81 (63–95)	<0.001
Preoperative renal Doppler ultrasonography			
Renal Doppler resistive index	0.68 (0.60–0.73)	0.64 (0.57–0.68)	0.027
Intrarenal venous flow pattern continuous, n (%)	6 (11)	21 (18)	0.205
Intrarenal venous flow pattern biphasic, n (%)	30 (55)	68 (58)	0.462
Intrarenal venous flow pattern monophasic, n (%)	19 (34)	29 (24)	0.836
Postoperative renal Doppler ultrasonography			
Renal Doppler resistive index	0.73 (0.67–0.75)	0.67 (0.62–0.73)	<0.001
Intrarenal venous flow pattern continuous, n (%)	4 (7)	19 (16)	0.170
Intrarenal venous flow pattern biphasic, n (%)	29 (53)	72 (61)	<0.001
Intrarenal venous flow pattern monophasic, n (%)	22 (40)	27 (23)	<0.001
Worsened intrarenal venous flow pattern, n (%)	32 (58)	22 (19)	<0.001
Postoperative inferior vena cava ultrasonography			
Maximum diameter (mm)	2.4 (2.2–2.6)	2.4 (2.1–2.6)	0.562
Collapsibility Index (%)	0.14 (0.09–0.16)	0.13 (0.09–0.21)	0.750
Renal Doppler ultrasonography at hospital discharge			
Renal Doppler resistive index	0.66 (0.60–0.71)	0.66 (0.58–0.70)	0.915
Intrarenal venous flow pattern continuous, n (%)	8 (14)	24 (20)	0.478
Intrarenal venous flow pattern biphasic, n (%)	36 (66)	80 (68)	0.554
Intrarenal venous flow pattern monophasic, n (%)	11 (20)	14 (12)	0.430
Outcomes			
Hospital length of stay (days)	5 (4–8)	4 (3–5)	0.003
Clavien–Dindo	1 (0–1)	0 (0–0)	<0.001

ACEI, angiotensin-converting enzyme inhibitor; ARB, angiotensin receptor blocker; MAP, mean arterial pressure; eGFR, estimated glomerular filtration rate; values are numbers (n), percentages (%) median and interquartile ranges (IQR) unless otherwise noted.

**Table 3:** Predictors of postoperative AKI by the Cox proportional hazard model.

Variables	B	HR	CI	*p*	B	HR	CI		*p*
Postoperative monophasic IRVF	2.950	19.108	4.845–75.359	<0.001	2.211	9.126	3.474–23.973		<0.001
Age (years)	−0.017	0.983	0.943–1.026	0.430					
Active smoker	−0.029	1.030	0.216–4.900	0.971					
Sex	0.039	1.040	0.219–4.924	0.961					
Preoperative RDRI	−4.045	0.018	0.000–9.096	0.205					
Intra-abdominal pressure	0.083	1.087	0.745–1.586	0.665					
Postoperative RDRI	8.206	33.314	0.025–23.376	0.046	7.235	13.925	1.583–12.420		0.036
Preoperative monophasic IRVF	−0.891	0.410	0.138–1.220	0.109					
Preoperative creatinine	1.338	3.812	0.238–60.953	0.344					
Charlson index	0.317	1.373	0.952–1.980	0.090	0.166	1.181	0.978–1.425		0.084
Arterial hypertension	−0.353	0.703	0.200–2.470	0.582					
Cadiovascular diseases	0.342	1.408	0.369–5.366	0.617					
Chronic respiratory diseases	−0.172	0.842	0.210–3.374	0.808					
Diabetes	0.058	1.059	0.272–4.124	0.934					

RDRI, renal Doppler resistive index; IRVF, intrarenal venous flow patterns.

**Table 4:** Complications in the 173 included patients.

Patients with complications, n (%)	55 (32)
Number of complications, n	70
Types of complications, n	
Sepsis	14
Bleeding	7
Respiratory failure	3
Ileus	4
Acute kidney injury	37
Anastomotic leakage	3
Intraoperative complication	1
Surgical wound infection	1

**Table 5:** Comparison between patients eventually developing any postoperative complications at day 7 versus those who did not.

	Postoperative Complications Group (n = 55)	No Postoperative Complications Group (n = 118)	*p*
Demographics			
Age, y	68 (60–73)	68 (60–73)	0.888
Male, n (%)	49 (89)	108 (92)	0.585
Body mass index, kg/m^2^	25 (23–29)	25 (23–28)	0.862
Baseline clinical data			
Charlson comorbidity index	4 (3–5)	4 (2–5)	0.057
ECOG performance status scale	0 (0–0)	0(0–0)	0.522
ASA classification	2 (2–2)	2 (2–2)	0.290
Baseline Laboratory data			
Hemoglobin, g/dL	14 (13–15)	15 (13–15)	0.707
Sodium, mmol/L	140 (139–142)	140 (139–141)	0.575
Potassium, mmol/L	4.4 (4.1–4.6)	4.2 (4.0–4.4)	0.166
Urea, mg/dL	39 (31–48)	37 (30–44)	0.926
Serum creatinine, mg/dL	0.90 (0.90–1.10)	0.90 (0.80–1.10)	0.773
eGFR mL/min/1.73 m^2^	74 (63–94)	85 (67–104)	0.113
Comorbidities, n (%)			
Hypertension	25 (48)	53 (47)	0.608
Diabetes mellitus	7 (14)	8 (7)	0.238
Active smoker	3 (6)	19 (17)	0.083
Chronic cardiovascular diseases	9 (19)	12 (11)	0.200
Chronic Respiratory diseases	5 (11)	8 (7)	0.529
Maintenance therapies			
ACEI/ARB, n (%)	24 (44)	39 (33)	0.235
β-Blocker	14 (28)	21 (19)	0.215
Loop diuretic, n (%)	5 (10)	15 (13)	0.796
Intraoperative period			
MAP, mm Hg	77 (70–85)	79 (75–85)	0.143
Heart rate, beats/min	60 (51–66)	61 (53–65)	0.651
Intra-abdominal pressure, mmHg	10 (10–12)	10 (8–12)	<0.001
Abdominal perfusion pressure, mmHg	66 (60–73)	69 (63–77)	0.030
Renal filtration gradient, mmHg	55 (50–61)	60 (54–66)	0.007
Length of surgery, minutes	150 (115–190)	135 (100–180)	0.402
Length of pneumoperitoneum, minutes	123 (66–150)	110 (73–150)	0.805
Trendelemburg, degrees (°)	20 (15–24)	20 (15–24)	0.430
Blood loss, mL	250 (50–500)	190 (48–300)	0.058
Intraoperative Fluids, mL	1400 (1100–1700)	1200 (838–1500)	0.059
Postoperative period			
Hemoglobin, g/dL	11 (10–13)	13 (11–13)	0.002
Sodium, mmol/L	139 (136–140)	139 (138–141)	0.740
Potassium, mmol/L	4.1 (3.9–4.5)	4.1 (3.8–4.3)	0.994
Urea, mg/dL	38 (29–48)	31 (25–39)	0.081
Postoperative Creatinine, mg/dL	1.4 (1.00–1.80)	1.0 (0.8–1.1)	<0.001
eGFR mL/min/1.73 m^2^	59 (38–82)	80 (63–95)	<0.001
Preoperative renal Doppler ultrasonography			
Renal Doppler resistive index	0.67 (0.59–0.70)	0.64 (0.57–0.68)	0.081
Intrarenal venous flow pattern continuous, n (%)	6 (11)	21 (18)	0.271
Intrarenal venous flow pattern biphasic, n (%)	30 (55)	68 (58)	0.743
Intrarenal venous flow pattern monophasic, n (%)	19 (34)	29 (24)	0.203
Postoperative renal Doppler ultrasonography			
Renal Doppler resistive index	0.74 (0.73–0.77)	0.67 (0.61–0.71)	<0.001
Intrarenal venous flow pattern continuous, n (%)	4 (7)	19 (16)	0.054
Intrarenal venous flow pattern biphasic, n (%)	29 (53)	72 (61)	0.324
Intrarenal venous flow pattern monophasic, n (%)	22 (40)	27 (23)	0.029
Worsened intrarenal venous flow pattern, n (%)	32 (58)	22 (19)	<0.001
Postoperative inferior vena cava ultrasonography			
Maximum diameter (mm)	2.4 (2.2–2.6)	2.4 (2.1–2.6)	0.102
Collapsibility Index (%)	0.14 (0.09–0.19)	0.13 (0.09–0.20)	0.828
Renal Doppler ultrasonography at hospital discharge			
Renal Doppler resistive index	0.66 (0.59–0.71)	0.65 (0.60–0.71)	0.886
Intrarenal venous flow pattern continuous, n (%)	8 (14)	24 (20)	0.407
Intrarenal venous flow pattern biphasic, n (%)	36 (66)	80 (68)	0.862
Intrarenal venous flow pattern monophasic, n (%)	11 (20)	14 (12)	0.169
Outcomes			
Hospital length of stay (days)	7 (4–9)	4 (3–5)	<0.001
Clavien-Dindo	1 (0–2)	0 (0–0)	<0.001

ACEI angiotensin-converting enzyme inhibitor; ARB, angiotensin receptor blocker; MAP, mean arterial pressure; eGFR, estimated glomerular filtration rate; values are numbers (n), percentages (%) median and interquartile ranges (IQR) unless otherwise noted.

**Table 6:** Predictors of postoperative any complications by the Cox proportional hazard model.

Variables	B	HR	CI	*p*	B	HR	CI		*p*
Postoperative monophasic IRVF	3.029	20.671	6.093–70.127	0.001	2.095	8.124	3.542–18.634		<0.001
Age (years)	−0.083	0.920	0.852–0.993	0.033	−0.021	0.135	0.953–1.006		0.135
Active smoker	−1.087	0.337	0.061–1.880	0.215					
Sex	1.149	3.155	0.730–13.630	0.124					
Preoperative RDRI	−2.511	0.081	0.000–28.887	0.402					
Intra-abdominal pressure	0.108	1.114	0.766–1.619	0.572					
Postoperative RDRI	1.414	4.113	0.813–20.812	0.087	1.096	2.993	1.522–5.884		0.001
Preoperative monophasic IRVF	−0.608	0.544	0.442–1.447	0.223					
Preoperative creatinine	0.904	2.468	0.205–25.807	0.451					
Charlson index	0.528	1.695	1.087–2.644	0.020	0.104	1.109	0.939–1.311		0.223
Arterial hypertension	0.883	2.417	0.758–7.711	0.136					
Cadiovascular diseases	−0.431	0.650	0.166–2.537	0.535					
Chronic respiratory diseases	−0.112	0.894	0.292–2.736	0.845					
Diabetes	−0.834	0.434	0.128–1.479	0.182					

RDRI, renal Doppler resistive index; IRVF, intrarenal venous flow patterns.

**Table 7:** Intraoperative characteristics of patients eventually developing monophasic IRVF patterns in the postoperative period compared with those who did not.

Variables	Postoperative Monophasic IRVF Pattern (71)	Postoperative Continuous or Biphasic IRVF Pattern (102)	*p*
MAP, mm Hg	76 (70–85)	79 (75–85)	0.358
Heart rate, beats/min	59 (51–66)	62 (54–65)	0.241
Intra-abdominal pressure, mmHg	10 (10–12)	10 (8–11)	0.016
Abdominal perfusion pressure, mmHg	66 (60–75)	69 (64–76)	0.246
Renal filtration gradient, mmHg	55 (50–63)	60 (54–65)	0.057
Length of surgery, minutes	144 (99–170)	140 (110–180)	0.920
Length of pneumoperitoneum, minutes	120 (65–145)	114 (83–163)	0.807
Trendelemburg, degrees (°)	20 (15–24)	20 (15–24)	0.257
Blood loss, mL	190 (48–313)	200 (80–400)	0.757
Intraoperative Fluids, mL	1500 (1200–1700)	1150 (800–1500)	0.029

**Table 8:** Multiple logistic regression analysis for parameters, predictors of monophasic IRVF pattern in the postoperative period.

Variables	B	OR	CI		*p*
MAP, mm Hg	−0.036	0.965	0.918	1.014	0.160
Heart rate, beats/min	−0.023	0.977	0.929	1.028	0.375
Intra-abdominal pressure, mmHg	0.303	1.354	1.017	1.804	0.038
Length of surgery, minutes	−0.002	0.998	0.973	1.025	0.898
Length of pneumoperitoneum, minutes	0.002	1.002	0.993	1.012	0.657
Trendelemburg, degrees (°)	−0.015	0.985	0.929	1.044	0.603
Blood loss, mL	0.001	1.001	0.999	1.003	0.372
Intraoperative fluids, mL	0.001	1.001	1.000	1.002	0.248

**Table 9:** Groups of patients divided according to arterial perfusion and venous congestion profiles assessed by Doppler ultrasonography.

Venous Pattern	RDRI	Number of Patients n, (%)	Patients with AKI n, (%)	Patients with any Complications n, (%)	Profiles Interpretation
Continuous	<0.72	13 (7.5)	0 (0)	0 (0)	Normal
	>0.72	9 (5)	2 (22)	3 (33)	Pure hypo-perfused
Biphasic	<0.72	62 (36)	2 (3)	3 (5)	Pure congestive compensated
	>0.72	18 (10.5)	3 (17)	5 (28)	Mild mixed hypo-perfused and congestive
Monophasic	<0.72	33 (19)	12 (36)	15 (46)	Severe congestive
	>0.72	38 (22)	18 (47)	29 (76)	Mixed severe hypo-perfused and congestive

## Data Availability

Data will be made available by the corresponding author for global collaboration on reasonable request, within the national restrictions imposed by privacy laws and ethics.

## Supplementary Materials

The following supporting information can be downloaded at: https://www.mdpi.com/article/10.3390/jcm12155013/s1, Figure S1: Flow diagram of the study.
