# Supplementary figures and images for: Perioperative Renal Ultrasonography of Arterio-to-Venous Coupling Predicts Postoperative Complications after Major Laparoscopic Urologic Surgery
Source: J Clin Med. 2023 Jul 30;12(15):5013. doi: 10.3390/jcm12155013 (PMC10419452; doi:10.3390/jcm12155013)

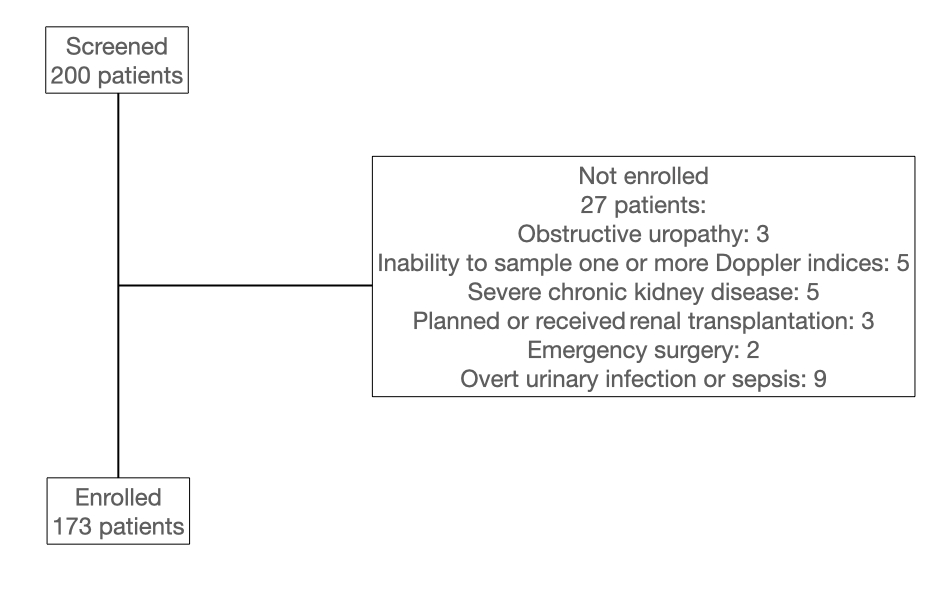

Supplement: Supplementary file 1 [file jcm-12-05013-s001.zip › jcm-2457100-supplementary/Figure S1.jpeg]
